# An 11-year-old boy with long lasting headaches

**DOI:** 10.1186/1546-0096-9-S1-P87

**Published:** 2011-09-14

**Authors:** Olga Vougiouka, Venetia-Maria Vraila, Lydia Kossiva, Dimitra Kallinikou, Dimitrios Kafetzis

**Affiliations:** 1 2nd Department of Paediatrics, Athens University Medical School – Paediatric Rheumatology Outpatient Clinic, Greece

## Aim

We report the case of an 11-year-old boy with headaches since 2 years

## Materials

The boy was referred to our department with right retrobulbal pain and bilateral papilledema, while brain MRI was normal. Physical examination revealed faint left radial pulse, non palpable left femoral artery, and hypertension, with right hand blood pressure 162/64 mmHg, in a difference with left hand blood pressure 128/70 mmHg. Bruits were auscultated all over the chest (precordium, supraclavicular, axillary and scapular area).

## Results

ECG and heart u/s were normal, but catheterization revealed stenosis of both pulmonary arteries, left subclavian artery, and high blood pressure.

CT-angiography demonstrated stenosis of aorta and its main branches, as well as both of renal arteries. The findings were compatible to Takayasu vasculitis. MAG3 showed left renal low perfusion index.

Cerebral CT and MRI, chest X-ray and non-specific blood tests were normal. Laboratory markers of inflammation and autoantibodies were absent.

The patient treated with prednisolone, azathioprime, ramiprile, isradipine and low dose aspirin. He remains in good clinical condition, free of headaches, and normal blood pressure.

## Discussion

Takayasu’s Arteritis (TA) is a chronic, large vessel systemic giant-cell arteritis, which is rarely reported in children. Pediatric patients may present with non specific symptoms, such as fever, weight loss, fatigue, and abdominal pain, or the disease may progress subclinically with unidentified headaches. EULAR/PRES criteria help to confirm the diagnosis. The prognosis is severe and unpredictable. It is therefore important to have high index of suspicion when dealing with a child with headache and hypertension. Blood pressure should be measured on both upper and lower extremities in cases of hypertension. Depending on the disease stage, systemic treatment and/or local surgical intervention are of benefit to the patient.

